# Human–Cougar interactions in the wildland–urban interface of Colorado's front range

**DOI:** 10.1002/ece3.5559

**Published:** 2019-08-20

**Authors:** Mathew W. Alldredge, Frances E. Buderman, Kevin A. Blecha

**Affiliations:** ^1^ Colorado Parks and Wildlife Fort Collins CO USA; ^2^ Colorado State University Fort Collins CO USA; ^3^ Colorado Parks and Wildlife Gunnison CO USA

**Keywords:** aversive conditioning, Colorado, conflict, Cougar, domestic predation, human interaction, livestock, predation, *Puma concolor*, residential development, wildland–urban interface

## Abstract

As human populations continue to expand across the world, the need to understand and manage wildlife populations within the wildland**–**urban interface is becoming commonplace. This is especially true for large carnivores as these species are not always tolerated by the public and can pose a risk to human safety. Unfortunately, information on wildlife species within the wildland**–**urban interface is sparse, and knowledge from wildland ecosystems does not always translate well to human‐dominated systems. Across western North America, cougars (*Puma concolor*) are routinely utilizing wildland**–**urban habitats while human use of these areas for homes and recreation is increasing. From 2007 to 2015, we studied cougar resource selection, human–cougar interaction, and cougar conflict management within the wildland**–**urban landscape of the northern Front Range in Colorado, USA. Resource selection of cougars within this landscape was typical of cougars in more remote settings but cougar interactions with humans tended to occur in locations cougars typically selected against, especially those in proximity to human structures. Within higher housing density areas, 83% of cougar use occurred at night, suggesting cougars generally avoided human activity by partitioning time. Only 24% of monitored cougars were reported for some type of conflict behavior but 39% of cougars sampled during feeding site investigations of GPS collar data were found to consume domestic prey items. Aversive conditioning was difficult to implement and generally ineffective for altering cougar behaviors but was thought to potentially have long‐term benefits of reinforcing fear of humans in cougars within human‐dominated areas experiencing little cougar hunting pressure. Cougars are able to exploit wildland**–**urban landscapes effectively, and conflict is relatively uncommon compared with the proportion of cougar use. Individual characteristics and behaviors of cougars within these areas are highly varied; therefore, conflict management is unique to each situation and should target individual behaviors. The ability of individual cougars to learn to exploit these environments with minimal human–cougar interactions suggests that maintaining older age structures, especially females, and providing a matrix of habitats, including large connected open‐space areas, would be beneficial to cougars and effectively reduce the potential for conflict.

## Graphical abstract

This manuscript summarizes cougar–human interactions and cougar use of exurban landscapes along the Front Range of Colorado. In this manuscript, we show how cougars are using the urban–wildland interface, investigate where interactions are occurring, and discuss management options to mitigate conflict.

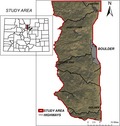

## 1 INTRODUCTION

Human beings dominate the world, and their influences on ecosystems and wildlife populations are well documented (Ellis, Goldewijk, Siebert, Lightman, & Ramankutty, 2010; Pickett et al., 2001; Theobald, 2005; Vitousek, Mooney, Lubchenco, & Melillo, 1997), but the impact on wildlife communities in the wildland**–**urban interface is not well understood, especially with regard to carnivores (Kertson, Spencer, Marzluff, Hepinstall‐Cymerman, & Grue, 2011). Population viability may be threatened by human development for some wildlife species (Hansen et al., 2005), but in other cases human development may result in ecosystem alterations that are beneficial to other species (Blecha, Boone, & Alldredge, 2018; Gehrt, Riley, & Cypher, 2010). Cougars likely represent the nexus of these two forces, as the wildland**–**urban interface represents a riskier environment where survival is typically lower, but also represents a beneficial environment with alternate and consistent prey resources (Blecha et al., 2018; Moss, Alldredge, & Pauli, 2016).

Carnivores are unique among wildlife as they tend to be highly adaptable and in general can exploit food resources within the wildland**–**urban interface, but they also generate considerable public attention with regard to human safety (Gehrt et al., 2010). The ability of carnivores to exploit these wildland**–**urban interfaces has been documented for cougars (Burdett et al., 2010; Kertson, Spencer, & Grue, 2013), bobcats (*Lynx rufus*; Donovan et al., 2011), black bears (*Ursus americanus*; Baruch‐Mordo et al., 2014, Lewis et al., 2015), and coyotes (*Canis latrans*; Gehrt, Anchor, & White, 2009, Poessel, Breck, & Gese, 2016). Unfortunately, as carnivores utilize the wildland**–**urban interface, and prey species increasingly inhabit urban areas, human–carnivore interactions will occur.

Understanding cougar–human interactions requires an understanding of the historical treatment of cougars throughout the history of human settlement in North America. Cougars once occupied the majority of North and South America, inhabiting most habitats from Northern British Columbia, south to Patagonia and from the Pacific to the Atlantic oceans (Rabinowitz, 2010). As North America was settled, prime cougar habitat and prey were lost and persecution of cougars began as livestock were killed and public perceptions of cougars villainized. Throughout much of the 20th century, a bounty was paid for cougars and they were killed wantonly across North America and completely removed from much of their historic range (Rabinowitz, 2010). Even in the western states where cougars persisted, numbers were greatly reduced.

Colorado protected cougars and began to actively manage for the sustainability of cougar populations in 1965, and by 1973, almost all of the western states were managing for cougar populations. Presumably, cougar populations began to increase and possibly reoccupy historic ranges following this protection (Anderson, Lindzey, Knopff, Jalkotzy, & Boyce, 2010). Coinciding with this was the rapid expansion of human populations. In Colorado, much of this expansion occurred in mountainous regions that were prime cougar habitat. Expanding human and cougar populations throughout the West have inevitably led to increasing cougar–human interactions in the late 1900s and early 2000s (Beier, Riley, & Sauvajot, 2010), including in Colorado (Halfpenny, Sanders, & McGrath, 1991). This significant increase in interaction during this time period led Colorado to initiate research to better understand this dynamic.

Knowledge of cougar space and resource use in the wildland**–**urban interface is limited. Other research has documented decreased use of areas with increased human density (Beier & Barrett, 1993; Burdett et al., 2010; Dickson & Beier, 2002), but this does not address how cougars use these areas. Kertson, Spencer, Marzluff, et al. (2011) examined space use of cougars in Western Washington within a wildland**–**urban interface and found that space use and movement rates within the urban areas were similar to those in wildland areas. They further suggest that the probability of human–cougar interactions is maximized at a threshold residential density that modifies available habitat but maintains enough wildland habitat to encourage moderate levels of cougar use. In the Santa Cruz Mountains of California, neighborhoods were found to be a deterrent to cougars for all behaviors or activities (Wilmers et al., 2013). Movement rates of cougars have also been shown to increase with increasing human density (Buderman, Hooten, Alldredge, Hanks, & Ivan, 2018; Wang, Smith, & Wilmers, 2017). Several studies have documented an increase in use of smaller‐bodied prey, synanthropic wildlife, and domestic pets in wildland**–**urban areas (Kertson, Spencer, Marzluff, et al., 2011), including two studies on the Front Range of Colorado (Blecha et al., 2018; Moss et al., 2016). Blecha et al. (2018) suggested that the utilization of higher housing density areas and, by extension, use of smaller‐bodied prey by cougars, is directly related to the length of time since a cougar last fed. However, there is much more to be learned about cougar space use and movement patterns within the wildland**–**urban interface. This includes the timing of use, where human–cougar interactions occur relative to how cougars and humans use the landscape, and management prescriptions to mitigate these interactions.

In general, ungulates are the primary prey for cougars (Iriarte, Franklin, Johnson, & Redford,^1990^; Murphy & Ruth, 2010), except in localities near housing development in which the cougar's dietary composition shifts to a higher reliance on smaller‐bodied prey (Kertson Spencer & Grue, 2011; Moss et al., 2016; Smith, Wang, & Wilmers,^2016^). Many synanthropic wildlife species, such as raccoons (*Procyon lotor*), and domestic animals are found at higher densities in developed areas (Bateman & Fleming, 2012), which may explain this switch to smaller‐bodied prey items, as they represent a consistent and abundant food resource. The use of alternate prey species within these developed areas also includes the use of domestic animals, including hobby livestock, dogs (*Canis familiarus*), and cats (*Felis catus*). The use of domestic animals by cougars has a high potential to be a source of human–cougar conflict, especially livestock as this is usually detected by owners compared with pets that just go missing. In addition, the state of Colorado is statutorily responsible for livestock damage from cougars ([Link] 33‐3‐104) and therefore must monetarily compensate owners when cougars take livestock. Understanding how cougars utilize domestic animals and whether cougars are habituated to preying on domestics or if this is opportunistic feeding is important for understanding the wildland**–**urban dynamic for cougars.

Although cougar attacks on humans are rare (Apker, Updike, & Holdermann,^2011^; CMGWG, 2005), they have increased in recent decades (Beier, 1991; Fithzugh, Kenyon, & Etling, 2003). There have been 3 human fatalities from cougars and 16 nonfatal attacks in Colorado since 1990 (CPW 2018, Reported lion attacks on humans). Along with this has been a concomitant increase in other human–cougar interactions, which are likely due to habitat reduction, human encroachment, increased human recreational activities, and possible increases in cougar populations (CMGWG, 2005). Studies examining the intrinsic factors leading to cougar–human interactions have provided inconsistent results due to geography, methodology, and data quality. Cougar–human interaction may be more likely in subadults (Aune, 1991; Mattson, 2007; Ruth, 1991) or males (Tiechman, Cristescu, & Nielsen, 2013), especially when involving livestock depredations (Aune, 1991; Shaw, 1977; Suminski, 1982; Torres, Mansfield, Foley, Lupo, & Brinkhaus, 1996). Analysis of conflict‐related mortalities in telemetered cougars has suspected higher likelihoods of conflict in male cougars (Thompson, Jenks, & Fecske, 2014) or in subadult dispersing males and the oldest cougars (Stoner, 2011). However, no significant age association was found when examining a large sample of necropsied cougar involved in pet and livestock depredations (Torres et al., 1996). Other analyses have suggested female cougars having a higher propensity to attack humans (Mattson, 2007). The CMGWG (2005) concluded that a combination of inexperience and unfamiliarity with their environment, as well as hunger, may cause young cougars to have more negative interactions with humans.

Wildlife managers throughout the western states, including those with Colorado Parks and Wildlife (CPW), are faced with decisions about how to manage cougar populations and individual cougars in order to maintain viable populations while also maintaining human safety. Defining acceptable levels of human safety is difficult because people's perceptions are different when interactions do not directly affect them. Other difficulties associated with managing cougar populations in areas with high levels of human interaction are caused by the limited amount of information that is currently known about cougars in these exurban situations and responses of cougars to management prescriptions (CMGWG, 2005).

Management plans generally require the removal of cougars that represent a danger to human health and safety, but the appropriate management response to cougars that are overly familiar or habituated to humans is unclear. Lethal control is losing public support (Reiter, Brunson, & Schmidt, 1999), so other options need to be examined. Shivik and Martin (2000) and van Eeden et al. (2018) emphasize the need to research and determine effective nonlethal control techniques, or managers risk losing credibility with the public.

We define aversive conditioning as applying a negative stimulus when an animal exhibits an undesirable behavior in an attempt to modify or abolish that behavior. Aversive conditioning has been attempted for a variety of species, including black bears, with mixed results (Beckman, Lackey, & Berger, 2004; Homstol, 2011; Leigh, 2007; Mazur, 2010; Rauer, Kaczensky, & Knauer, 2003). In these examples, specific behaviors are generally targeted, such as the use of trash or dumpsters by bears. McCarthy and Seavoy (1994) conclude that aversive conditioning for bears may be useful where single anthropogenic food sources occur, but are questionable in urban areas where resources are widely distributed.

There have been no studies confirming the effectiveness of aversive conditioning in cougars (CMGWG, 2005). Beier (1991) describes two unsuccessful attempts at aversive conditioning (one shot with rock salt, one treed and collared); however, one of these cougars was already exhibiting aggressive behavior and the other was in poor condition. McBride, Jansen, McBride, and Schulze (2005) used hound capture and subsequent hound chases as a form of aversive conditioning on 4 Florida panthers with some degree of success. However, in Central Mexico, visual and sound deterrents were effectively used to scare felid predators away from livestock (Zarco‐Gonzalez & Monroy‐Vilchis, 2014). Aversive conditioning may be particularly difficult with cougars, as they are preying on naturally occurring prey species in urban areas (deer, raccoons etc.), as well as on pets and livestock. In many cases, the undesirable behavior is not associated with the prey species, but rather the location where they are hunting and making kills. Therefore, associating a negative stimulus with an undesirable behavior (location) may be particularly difficult or impossible for cougars compared with aversive conditioning on other species where there is a single source to condition against.

Large carnivores in the wildland**–**urban interface worldwide are often at risk because their requirements may conflict with those of humans causing an urgent need for techniques to resolve and understand conflicts between people and predators (Woodroffe, 2000). To grow the knowledge base of how to potentially manage these conflicts, we examined cougar spatio‐temporal landscape utilization patterns, prey utilization, and implementation of a potential conflict management techniques in an urban–wildland interface system in the Colorado Front Range. Specifically, we model the habitat selection of a representative sample of GPS‐telemetered cougars and compare this to where sightings, conflicts, and harvest occurred as reported by agency personnel. We put the cougar landscape selection and conflict event patterns into context by summarizing the true usage patterns and quantity of domestic prey species killed by the sample of telemetered cougars on the same landscape. Along with these population‐wide data, we present case studies of individual cougars with regard to conflict to address habitual versus opportunistic behavioral responses. Finally, we address the feasibility and effectiveness of aversive conditioning techniques on cougars and how this applies to future management of cougars within the urban–wildland interface. These results are likely applicable to many felid species inhabiting such areas and may be broadly applicable to carnivore species in urban settings.

## 2 STUDY AREA

**Figure 1:**
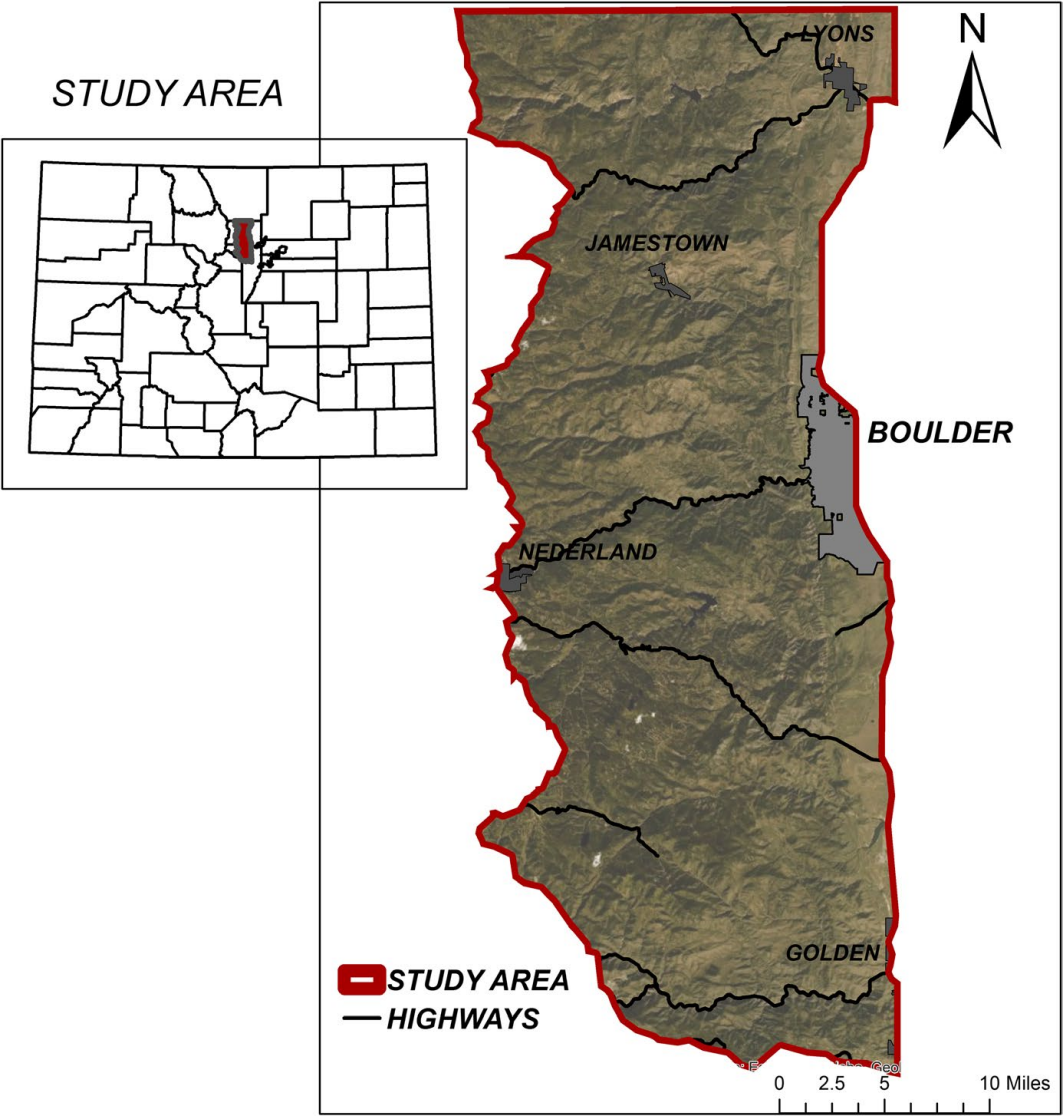
Study area used to investigate human–cougar interactions along the Front Range of Colorado

This study was part of a long‐term cougar study (2007–2015) conducted in Colorado's northern Front Range including Boulder, Jefferson, Gilpin, Clear Creek, and Larimer counties (Figure 1). This is a foothill–montane system covering an elevation gradient from 1,590 m along the urban eastern edge to 3,170 m along the wildland western edge approaching the continental divide. Housing density across the study area included wildland/rural (0–0.068 houses/ha, 69.7%), exurban (0.068–1.47 houses/ha), suburban (1.47–10 houses/ha, 2%), and urban (>10 houses/ha, 3%) (Blecha, 2015; Theobald, 2005), creating a patchwork of habitats across the study, with the majority of urban areas along the eastern edge. Cities and counties throughout the area have also purchased and maintain large parcels of land as open space managed for human recreation and wildlife populations. Naturally occurring prey species within this study area include elk (*Cervus canadensis*), mule deer (*Odocoileus hemionus*), cottontail rabbit (*Sylvilagus nuttallii*), raccoon (*Procyon lotor*), and striped skunk (*Mephitis mephitis*). For detailed study area descriptions, see Moss et al. (2016) and Blecha et al. (2018). The majority of livestock in the area was hobby livestock but will be referred generally as livestock throughout.

## 3 METHODS

### 3.1 Cougar capture and incident data

Cougars were captured from 2007 to 2015 (2014 and 15 to remove collars) using hounds, cage traps, foothold snares, and free‐darting, and immobilized using medetomidine and ketamine hydrochloride. Female cougars > 12 months old and male cougars > 24 months old and >55 kg were collared with satellite GPS collars (Vectronics, GPS Plus Globalstar). Ear tag transmitters (ATS VHF, 28 g) or ARGOS Satellite, 28 g) was used for cougars too small to be collared. Age was estimated using gum‐line recession or date of birth, animals were weighed, morphometric measurements were taken, and blood and tissue samples were collected for all individuals captured. All capture and handling was done under approved capture and handling guidelines (ACUC 01‐2007 and 16‐2008). For the duration of the study, GPS collars were set to collect 7 to 8 locations per day (maintaining a schedule at night of every 3 hr and reducing locations during the day when only collecting 7 per day). See Blecha and Alldredge (2015) for detailed description of capture, handling, and GPS collar settings.

Cougar incident data between 2001 and 2014 within the northern Front Range of Colorado were collected and summarized by event type and location. When CPW is contacted about cougar sightings or conflicts, CPW personnel fill out and keep records on these events (known as “incident forms”). Data on these events, along with CPW's cougar harvest data, were summarized and used in analyses to determine site characteristics of where these events occurred. There were 629 conflict and sighting incidents reported from 2001 to 2014. We will refer to a direct human encounter with a cougar or property damage by a cougar as a conflict and a report that a cougar was seen in the area as a sighting. Collectively, we will refer to these as “cougar incidents.”

### 3.2 Timing of use and incident locations

Understanding the timing of when cougars use areas with different housing densities is important to understanding cougar behavior and potential management strategies. We examined four housing density classes aggregated at a 300 m scale; 0 houses per ha (wildland), 0 to 1.47 (rural and exurban), 1.47 to 10 (suburban), and greater than 10 houses per ha (urban). Hour of the GPS collar locations was discretized into four bins: night (22:00–05:00), morning (05:00–09:00), day (09:00–17:00), and evening (17:00–22:00) time periods. For each individual and time period, we summed the number of GPS collar location observations in each housing density class. To standardize the proportional use of the housing densities across time (because the time periods vary in duration), we then divided the resulting value by the total number of locations (for that individual) that fell into each time bin class.

To model the risk of cougar incidents, we employed a use‐availability framework fit using a logistic regression (Manly, McDonald, Thomas, McDonald, & Erickson, 2007). In the use‐availablility framework, covariates from locations where cougar incidents occurred (used) are contrasted with random locations selected from an area considered available for cougar incidents (available). In this application, available locations were restricted to GPS locations of collared cougars using a latent selection difference function given that availability locations can also be viewed as a used location during an alternative behavioral state (e.g., Erickson, Found‐Jackson, & Boyce, 2014; Latham, Latham, & Boyce, 2011; Lendrum et al., 2018; Roever, Beyer, Chase, & Aarde, 2014) as opposed to a random selection of locations throughout the study area or home range where the animal may or may not have been observed. In the literature, this particular application of the use‐availability framework has sometimes been referred to as using latent selection difference functions (LSD; e.g., Erickson et al., 2014; Latham et al., 2011; Lendrum et al., 2018; Roever et al., 2014). Typically, they are referred to as LSDs when the availability locations (represented by a 0 response in a logistic regression) represent something that can also be viewed as a used location (e.g., locations used by another species or during an alternative behavioral state), as opposed to a random selection of locations where the animal was not observed. Given this sampling scheme, we were able to assess the difference between where cougar incidents did and did not occur, conditioned on cougar presence.

A habitat selection model was necessary to visualize the relative risk of a cougar incidents occurring on the landscape. Because the cougar incidents analysis relied on selecting random available locations from used cougar locations, the cougar incident regression equation actually represents the relative risk of a cougar incident conditioned on cougar selection. Spatially interpolating this function assumes that cougar preference is uniform across the landscape. To account for this, we needed to multiply the relative risk of a cougar incident (conditioned on cougar selection) by relative cougar habitat selection. We therefore fit a resource selection function (RSF) assuming an exponential form and estimated the coefficients using a Bayesian hierarchical logistic regression (Johnson, Nielsen, Merrill, McDonald, & Boyce, 2006). The used sample for the cougar habitat selection model were the 5,000 randomly selected GPS points used as the available sample in the cougar incidents model, and 25,000 available points were sampled uniformly across the study area. Informal sensitivity tests were performed to determine that the number of used and available locations were appropriate. Housing density level was used as a factor predictor, but suburban and urban were combined due to the small number of urban grid cells. Continuous predictor variables included canopy presence, distance to canopy, heat loading (CHILI, Theobald, Harrison‐Atlas, Monahan, & Albano, 2015), elevation, distance to roads (Colorado Department of Transportation), distance to housing (Blecha, 2015), and topographic wetness (Theobald, 2007), all of which were standardized to the mean and standard deviation across the study area. We also used an additional interaction term between housing density and distance to housing. Due to high correlation among the covariates with the interaction term, we orthogonalized (a mathematical process that decorrelates sets of values) the covariates to fit the model and then back‐transformed the coefficients to be comparable to the results from the cougar incident analyses. We present the relative selection strength across the study area and the posterior mean and 95% credible intervals for the regression coefficients (e.g., the log relative selection strength of a given covariate, such that negative values indicate avoidance and positive values indicate preference; Avgar, Lele, Keim, & Boyce, 2017; Lele, Merrill, Keim, & Boyce, 2013). Our primary reason for fitting a habitat selection model was to help visualize the conditional relationship between cougar incidents and cougar use; therefore, we did not fit age or sex‐specific habitat selection models (for most conflicts, sex and age of the cougar were unknown).

In the cougar incident models, the conflict and sighting locations were considered the used sample, and a randomly selected subset of 5,000 cougar locations, regardless of the individual, was the available sample. Because the available sample in this analysis is related to what we define as the used sample in the habitat selection model described in the above paragraph, the computational load of using all 233,348 locations as the used sample (necessitating a minimum of 1,166,740 available locations given the conventional guidance on habitat selection analysis) was computationally infeasible. We used the same model specification for the cougar incident models as for habitat selection, but without the interaction between housing density and distance to housing. Without the interaction term, orthogonalization of covariates was not necessary.

For all models, we estimated coefficients using a Bayesian hierarchical logistic regression, which was fit in R (R Core Team, 2017) using a Gibbs sampler with adaptive tuning. Adaptive tuning occurred during the first 20,000 iterations; the final tuning coefficient was then used for a subsequent 20,000 iterations, with the first 2,000 iterations being discarded. Throughout we will use the terminology relative selection strength or relative risk when discussing coefficient estimates, as noted by Lele et al. (2013) and Avgar et al. (2017). We present both the estimated coefficients (e.g., the log relative risk of a given covariate) and a modified spatial description of the relative risk of a cougar incident on the landscape. To present the latter, we used the inverse logit of the estimated cougar incident regression equations, excluding the intercept, to visually describe the relative risk of a cougar incident and constrain the values between zero and one, and then multiplied each surface by a surface representing relative cougar habitat selection.

### 3.3 Sampled collared cougar case histories and domestic prey

In order to understand cougar incident behavior, we summarized the number of collared cougars that engaged in conflict behavior during the study and the types of behaviors by individual. Sample size is small and the types of conflict are varied, so detailed analyses were not warranted. We also believe that individual variation among cougars is important and would be masked by generalities about habituation or the lack of it. Therefore, we present all case histories on a time line showing the type of conflict and when it occurred for each individual in an attempt to demonstrate patterns of conflict among individuals. This summary includes only reported conflicts and does not include domestic kills documented during feeding site investigations (see below) that were not reported by owners as conflict.

Cougar feeding patterns on domestic prey were derived and summarized based on the field investigation of GPS location clusters (including single point clusters representing small prey) produced by GPS collared cougars (Anderson & Lindzey, 2006). A stratified random sample of potential feeding sites were ground‐truthed by project personnel and classified by feeding event presence, prey species, and whether the species was domestic, for 56 cougars. Sampling strata were based on each unique cougar and month monitored. See Blecha and Alldredge (2015) for detailed sampling and field methods. At the population level, we calculated an annual per‐capita average proportion and kill rate of domestic prey in the diet by accounting for the unequal sampling probability of clusters across monthly strata. Given that the number of kills cougar make, and therefore the corresponding number of clusters available to be sampled, is variable throughout the year (Knopff, Knopff, & Boyce, 2010, CPW unpublished data), we accounted for this unequal sampling probability across our monthly strata (R package: SURVEY). Sample coverage probability by month was calculated using the expected per‐capita feeding rate (*f*) multiplied by the number of unique combinations (*k*) of cougar and year monitored in each month, divided by the total kills sampled (*n*) per month. Confidence interval was calculated based on techniques for small expected proportions (Korn & Graubard, 1998). On an individual level, the total feeding events investigated, proportion of feeding events determined to be domestic, and the measure of effort expended (the number of months an individual cougar's clusters were investigated) were summarized for each collared cougar.

Finally, a generalized additive model (binomial error family) was used to test for variables associated with feeding on domestic prey over wild prey, given a cougar feeding event (domestic prey coded as 1 and wild prey coded as 0). Candidate models included all combination subsets of Julian calendar day number (1–365 days: cubic cyclic spline term), cougar age, cougar sex (male as baseline), and housing density (400 m buffer). Cougar age (by month) was determined dynamically, as some individuals were monitored over several years. Nonlinear or interaction responses were considered in housing density, cougar age, and cougar sex with simple smooth splines or simple quadratic terms. Models were evaluated via log likelihood, AICc_c_, and AICc weight.

### 3.4 Aversive conditioning

We also assessed the efficacy of aversive conditioning on cougars in urban areas by describing cougar responses to several different types of aversive conditioning. Aversive conditioning treatment was applied to an individual GPS collared cougar in an attempt to alter its behavior in the future. The primary behavior that we focused on was use of undesirable locations, such as urban neighborhoods and areas near schools. Research from this study has already demonstrated that cougars are generally using these areas to acquire food (Blecha et al., 2018), so aversive conditioning was typically applied to situations where a cougar had made a kill in an urban area. A similar situation would be a cougar that had sought cover (thick brush or under a porch) in urban areas during the day. A secondary situation for aversive conditioning is a cougar that had killed a domestic animal (livestock or pet). In Colorado, a livestock owner can request that a cougar be killed if it has killed livestock, so aversive conditioning in these situations was uncommon.

Aversive conditioning was not done in more remote areas or lower housing density exurban areas where cougars were preying on naturally occurring prey items, even if this occurred near housing or if the cougar was just seen and reported. These situations were viewed as natural behaviors for cougars and would not normally elicit a response from wildlife managers. All attempts of aversive conditioning were done for situations and reports that would normally result in wildlife managers hazing, trapping, or removing a cougar. All cougars that were aversively conditioned were either previously collared or were collared as part of the treatment so that responses could be assessed.

Initially aversive conditioning treatments involved shooting the offending individual with 2 to 3 bean bag rounds fired from a 12‐gauge shotgun at a distance > 10 m up to 30 m to avoid injury to the animal. Releases were always set so that the treatment could be consistently done by trained personnel. In many situations, the cougar was in an undesirable location, so capture and relocation were also necessary. In these cases, the cougar was free‐range darted or captured in a cage trap, relocated to a nearby open space 4–20 km away, and shot with bean bag rounds on release. Relocation distance was intentionally kept small in order to assess the effects of the aversive conditioning treatment on behavior rather than the effect of relocation. In the latter part of the study (2011–2014), based on responses to aversive conditioning, we assessed the response of cougars to removing their kills from undesirable locations. In these cases, GPS data were used to find kills in these areas, and then, kills were removed as soon as they were detected.

The types of human–cougar interactions were varied, most situations were unique, and sample sizes were small so we summarized these interactions into general categories including undesirable location (areas of high housing density, inside city limits, near school), undesirable kill location, and domestic predation. Aversive treatments were also unique to each situation; therefore, we present these data and cougar responses as summaries because the variability in each situation did not warrant statistical analysis.

## 4 RESULTS

### 4.1 Timing of use and incident locations

**Figure 2:**
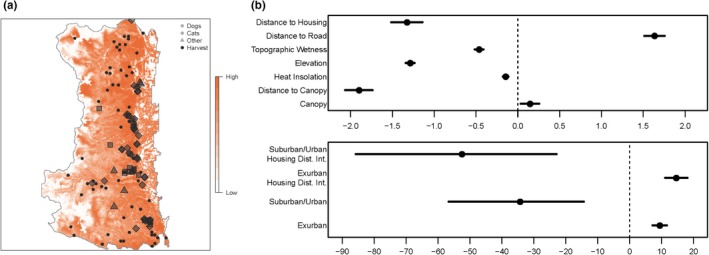
Relative probability of cougar use within the study area, given the covariates and their effect sizes, and the log relative selection strength of a given covariate, such that negative values indicate avoidance and positive values indicate preference. Black circles on the map indicate cougar harvest locations, while gray symbols indicate reported cougar predation on domestic animals and livestock. Suburban/Urban Housing Dist. Int. and Exurban Housing Dist. Int. represent the additional effect of distance to housing when an individual was in a suburban/urban area or exurban area, respectively (e.g., the interaction term between housing density and distance to housing)

We examined the timing of when cougars used various housing densities and where incidents occurred relative to how cougars used the landscape for 76 (43 females, 33 males) independent age cougars with GPS collars. Of these cougars, 6 females (14.0%) and 11 males (33.3%) never used areas with housing densities over 1.47 houses/ha (suburban/urban) and 32 females (74.4%) and 28 males (84.8%) never used areas with more than 10 houses/ha (urban). Cougar use of higher housing density areas was predominantly at night (Table 1). Eighty‐three percent of cougar use in areas exceeding 10 houses/ha and 62% of cougar use in areas between 1.47 and 10 houses/ha occurred between 22:00 and 05:00 hr. Cougars selected exurban over rural habitat (*β* = 9.45) and strongly avoided suburban/urban habitat relative to rural habitat (*β* = −34.29; Figure 2). Cougar's relative selection strength declined as a function of distance to housing; however, this effect describes the baseline cougar response to housing (in rural areas; *β* = −1.33; Figure 2). In exurban areas, there was an overall positive effect of distance to housing on selection (*β* = 14.63, for an overall effect of 13.33); however, in suburban and urban areas, cougars demonstrated strong selection with decreasing distance to housing (*β* = −52.51, for an overall effect of −53.84; Figure 2). Cougars showed a preference for areas further from roads (*β* = 1.64; Figure 2). The presence of canopy cover increased cougar preference (*β* = 0.15); however, cougars demonstrated avoidance of areas with increasing distance to canopy (*β* = −1.90), heat loading (*β* = −0.15), elevation (*β* = −1.29), and topographic wetness (*β* = −0.46; Figure 2).

**Table 1:** Timing of use within habitat density classifications relative to daily activity periods: night (22:00–05:00 hr), morning (05:00–09:00 hr), day (09:00–17:00 hr), and evening (17:00–22:00 hr)

Housing density (houses/ha)	Night	Morning	Day	Evening
0	0.22	0.27	0.28	0.24
0–1.47	0.40	0.17	0.14	0.29
1.47–10	0.62	0.06	0.05	0.27
>10	0.83	0.05	0.02	0.11

Note

Proportion use was standardized to account for differing GPS location schedules within time periods.

**Figure 3:**
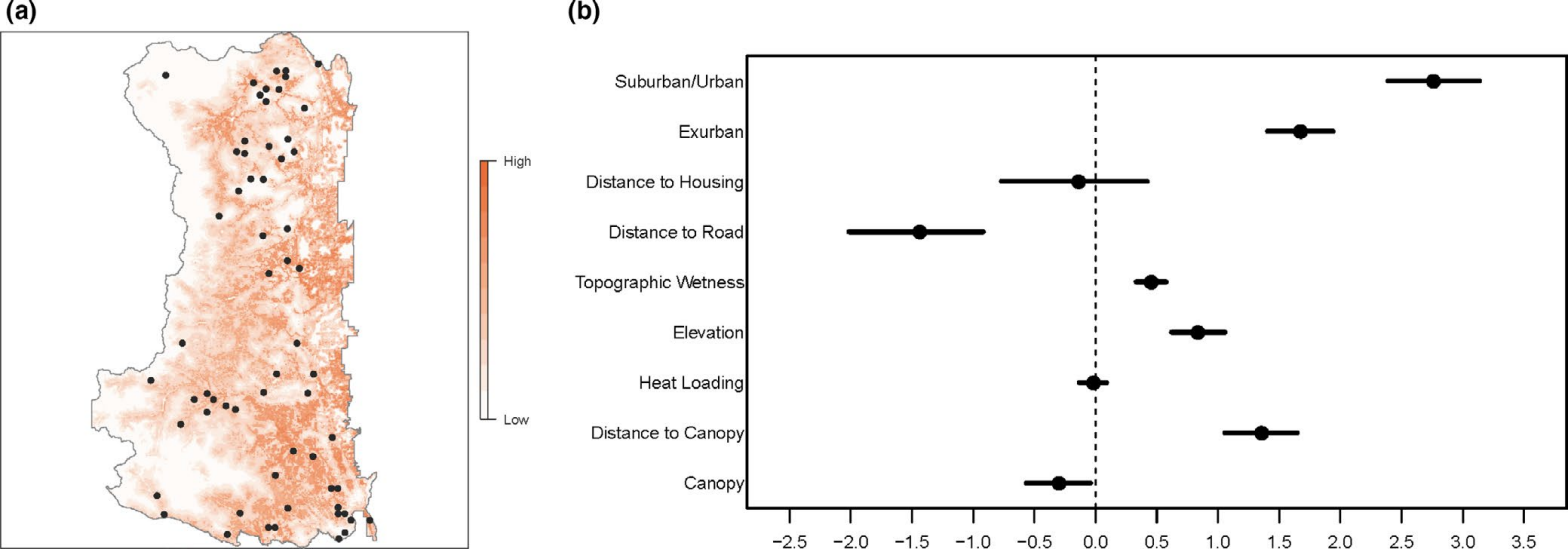
Relative risk of a reported cougar conflict event given the relative probability of cougar use within the study area, and the log relative risk strength of a given covariate. Negative values indicate decreased risk, and positive values indicate increased risk. Black circles on the map indicate cougar harvest locations

Conditional on where conflict is more likely to occur based on cougar selection models, the presence of canopy cover (*β* = −0.30) and increasing distance to roads (*β* = −1.45) decreased the relative risk of a reported conflict event (Figure 3). Increasing distance to canopy (*β* = 1.36), elevation (*β* = 0.84), and topographic wetness (*β* = 0.45) increased the relative risk of a reported conflict event (Figure 3). Being in an exurban (*β* = 1.67) or suburban/urban (*β* = 2.76) area also increased the relative risk of a reported conflict event, with the relative risk being higher in suburban/urban areas (Figure 3).

**Figure 4:**
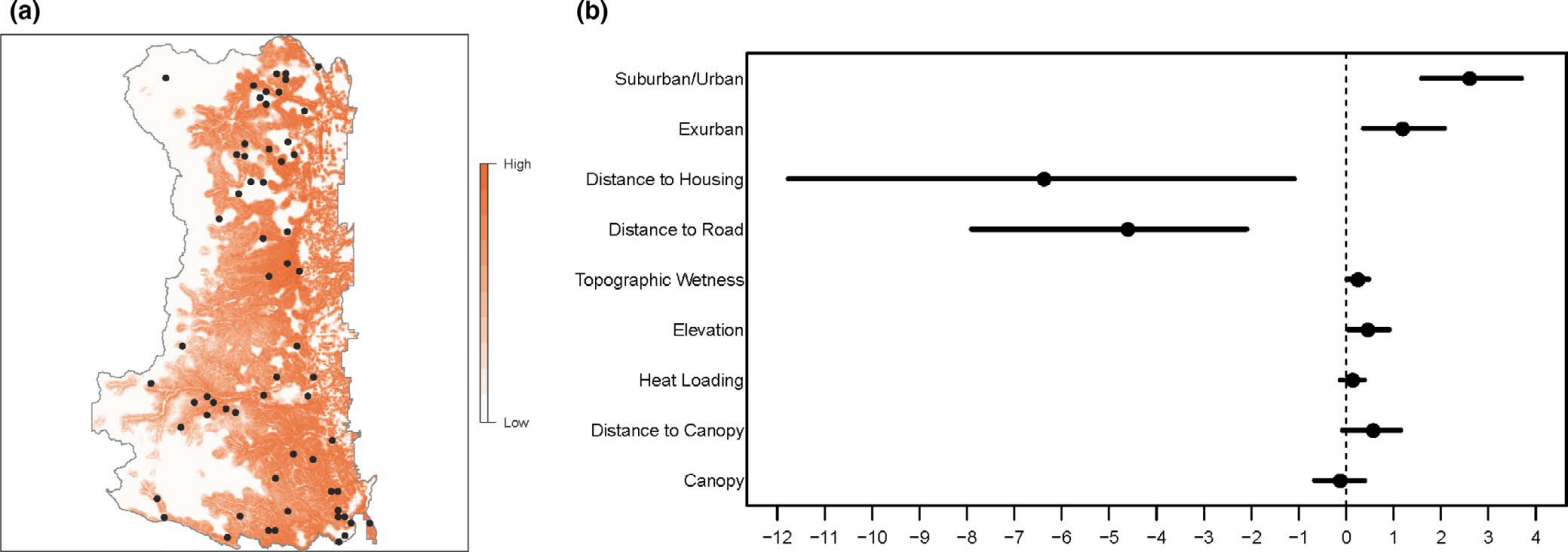
Relative risk of a cougar sighting event, given the relative probability of cougar use, within the study area, and the log relative risk strength of a given covariate, such that negative values indicate decreased risk and positive values indicate increased risk. Black circles on the map indicate cougar harvest locations

The relative risk of a sighting event is also implicitly conditioned on where human activities occur; however, this component was unobserved and unmodeled, and therefore, care should be taken when interpreting the effects of covariates on relative risk of a cougar sighting. Increasing elevation (*β* = 0.46) and topographic wetness (*β* = 0.24) had a positive effect on the relative risk of a cougar sighting, given cougar selection (Figure 4). Similar to conflict events, being in an exurban (*β* = 1.18) or suburban/urban area (*β* = 2.60) also increased the relative risk of a sighting event. Increasing distance to road (*β* = −4.61) and increasing distance to housing (−6.38) decreased the relative risk of a sighting event (Figure 4).

Spatially, the relative risk of a sighting event was similar to the relative risk of a conflict event, however less heterogeneous across the landscape, which is likely due to the nonsignificant effects of canopy, distance to canopy, heat loading, and the small positive effects of elevation and topographic wetness (Figure 4). Cougar predation on domestic animals generally occurred in areas that cougars were selecting for and within the higher housing density areas along the eastern edge of the study area (Figure 2). Some predation on livestock and dogs did occur in lower housing density areas in the western and central portions of the study area. Although some cougar harvest occurred in the higher housing density areas along the eastern edge of the study area, the majority was in lower housing density areas in the western and central portions (Figure 2). In general, cougar harvest did not occur in areas where cougars tended to be seen or in conflict with humans (Figure 3).

### 4.2 Cougar case histories and domestic prey

**Figure 5:**
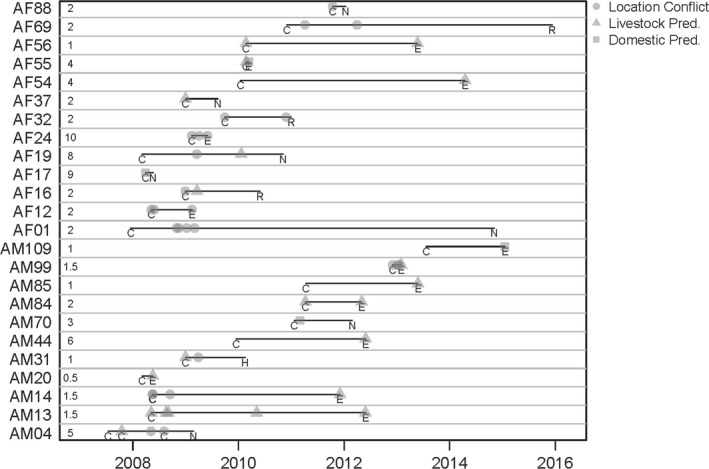
Timeline for monitored cougars involved in at least one human conflict event in the Front Range of Colorado from 2007 to 2016. Letters indicate a capture event (C), a mortality event (H: hunting, N: natural, E: euthanized), or collar removal (R). Symbols indicate a conflict event, which may have overlapped with a capture or mortality event. Values at the start of each row indicate age at first capture

Of 52 males and 50 females that were captured and monitored during the study, 11 males and 13 females were reported by the public for conflict behavior. Of the males, 5 were initially captured and collared because of conflict behavior (3 livestock predation and 2 because of location) (Figure 5). Of the females, 9 were initially captured and collared because of conflict behavior (3 livestock predation, 1 domestic pet predation, and 5 because of location). A total of 24 conflict events were reported for male cougars: 14 for livestock predation, 2 for predation on dogs, and 8 for using an undesirable location. AM13 was the only male that showed repeated livestock predation over a short time period, killing a llama (*Lama glama*) and 2 small horses over a 5‐month period but then going for several years before killing livestock again. AM14 never killed livestock until he was 6 years old and then was euthanized for killing multiple llamas in one area over a month. A total of 26 conflict events were reported for female cougars: 7 for livestock predation, 2 for predation on dogs, and 17 for using an undesirable location.

Domestic prey did not appear in kill sites for 34 of 56 cougars sampled in kill site investigations. Of all of the GPS clusters that were investigated, 1,625 were verified as kill sites, and of those, 68 contained domestic prey items: 43 cat, 17 dog, 2 alpaca, 2 goat, 1 sheep, 1 llama, 1 fowl, and 1 unidentifiable pet (based on the presence of a collar). Domestic species constituted 3.87% (95% C.I.: 2.87%–5.0%) of items preyed upon annually. Interindividual variation (by cougar) of domestic predation was present based on a nonuniform distribution in domestic prey proportion when considering each cougar as a single sample. Domestic prey proportion by individual ranged from 0 to 0.75 (Avg. 0.16, Table 2). However, the high proportion (0.75) of domestic prey was for a single individual that was only monitored for two months in the study (4 total kill sites documented). The individual with the next highest proportion of 0.357 was monitored for 15 months with 28 total kill sites documented. The proportion of domestic prey found at cougar feeding sites ranged from 0.01 in January to 0.10 in May (Table 3).

**Table 2:** Proportion of domestic prey found at cougar kill sites in relation to the number of kill sites investigated and the number of months kill sites were investigated, reported by cougar

Cougar ID	Months monitored	Events investigated	Proportion domestic
AF58	2	4	0.75
AF56	15	28	0.36
AF37	3	3	0.33
AM46	3	4	0.25
AM70	10	16	0.25
AM74	16	34	0.21
AF24	3	5	0.20
AM606	7	17	0.18
AM21	2	6	0.17
AF32	9	20	0.15
AF88	4	7	0.14
AF40	36	73	0.10
AM44	30	52	0.08
AF73	21	43	0.07
AF52	30	68	0.06
AF57	25	54	0.06
AF86	9	19	0.05
AM14	29	42	0.05
AF69	24	64	0.05
AM84	13	25	0.04
AF50	16	37	0.03
AF59	22	40	0.03

Note

Kill sites were investigated for 56 cougars but domestic prey was found at kill sites for only these 22 individuals.

**Table 3:** Observed monthly proportion of domestic prey found at kill sites (*p*_d_), per‐capita feeding rate based on all prey items (*f*_m_), number of cougars monitored each month across years (*k*_m_), and the number of ground‐truthed clusters confirmed as kill sites for all prey items (*n*_m_)

	*p*_d_	*f*_m_	*k*_m_	*n*_m_
January	0.01	6.09	70	152
February	0.06	5.76	62	115
March	0.04	5.68	65	139
April	0.05	6.72	68	149
May	0.10	6.39	64	134
June	0.04	11.31	66	138
July	0.03	12.51	63	173
August	0.05	9.91	65	144
September	0.01	10.78	61	127
October	0.06	10.43	58	111
November	0.02	7.92	64	124
December	0.03	8.01	58	119

**Figure 6:**
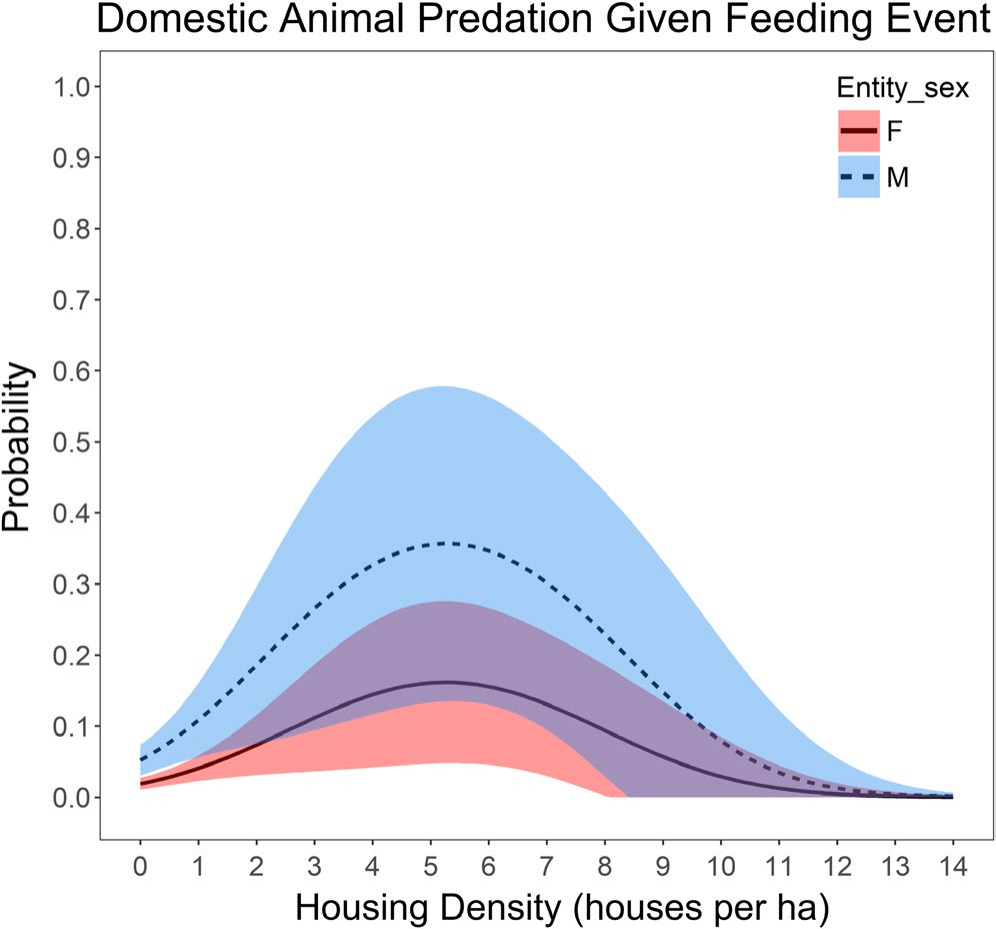
Model predicted domestic prey probability and 95% confidence intervals, given a feeding event, as a response to housing density for male and female cougars

**Figure 7:**
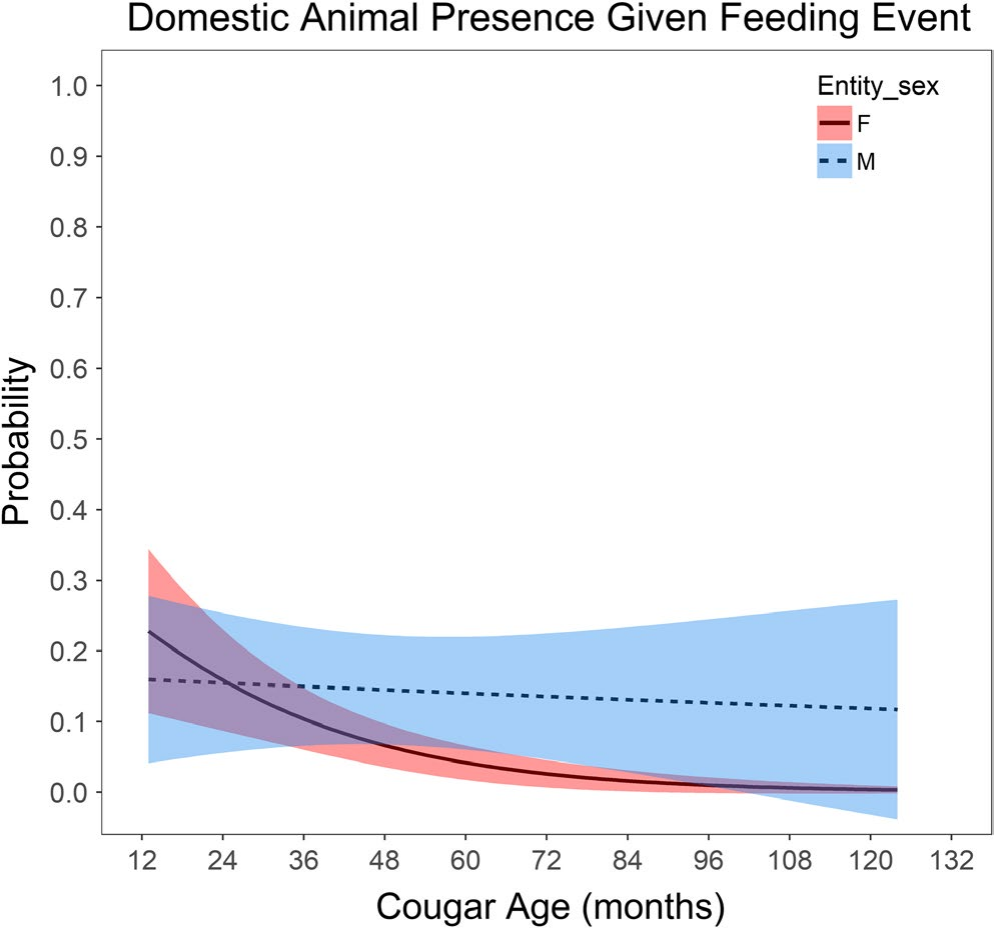
Model predicted domestic prey probability, given a feeding event, as a response to cougar age and sex as interacting variables. Housing density was held at 1.47 houses per ha, which is the transition point from exurban to suburban housing density

Conditional on a feeding event, domestic prey item presence was most parsimoniously described as a function of housing density with a quadratic (*β*_HD_ = 1.019 (*SE* = 0.246), *β*_HD_^2^ = −0.128 (*SE* = 0.042)), cougar age (*β*_age_ = −0.762 (*SE* = 0.179)), cougar sex (*β*_male_ = 0.479 (*SE* = 0.138)), and an interaction between sex and age (*β*_male,age_ = 0.387 (*SE* = 0.134)), holding 77.6% of the AIC_c_ weight. Julian calendar day appeared in the second‐ranked model holding 11.1% AIC_c_ weight. The influence of housing density on domestic prey killing probability was strongest at ~5 houses per ha (Figure 6). This influence of housing density appears to diminish with >5 houses per ha, but this declining response may be an artifact of the small sample size of feeding events in these highest housing densities (1.7% of all 1625 feeding sites). Cougar age and sex were interacting variables revealing that age of the individual did not affect the likelihood of domestic prey at a kill for males, but subadult female cougars were more likely than all other age–sex types to prey upon domestic animals (Figure 7).

### 4.3 Aversive conditioning

From 2007 to 2011, 7 female and 6 male cougars were aversively conditioned from 1 to 3 times each for a total of 17 and 8 aversive conditioning treatments on females and males, respectively. Aversive conditioning treatments for being in undesirable location were conducted 14 and 3 times for females and males, respectively, and all of these treatments included shooting the offending individual with 1 to 2 beanbag rounds. A naturally occurring food source was also involved in 16 of these cases. Because of the location, 15 aversive treatments for undesirable location also included relocation to nearby open space. Relocation distances between 4 and 9 km (*n* = 13) resulted in the cougars return within 1 to 2 days post‐treatment. Relocation distances of 16 and 19 km for the remaining 2 cougars resulted in a return to the individual's home range within 6 days, but no immediate return to the location of conflict. All females continued to use locations of potential conflict (neighborhoods within city limits) after treatments. Only 1 male continued to use these areas after treatment; however, 2 males were later aversively conditioned for killing livestock.

The remaining 8 aversive conditioning treatments involved cougars killing livestock (*n* = 5) or domestic dogs (*n* = 3). In four cases, aversive conditioning was done on site and did not involve capture or relocation, while the remaining 4 were relocated. All relocated cougars returned to the capture location within 2 days, but immediately left the area, presumably due to prey carcass removal. Two cougars never killed livestock again post‐treatment and 2 were euthanized for killing livestock within 6 months following treatment. The remaining cougars opportunistically killed livestock for the rest of their lives (4 to 6 additional years each making 1 to 2 total livestock kills) and were eventually euthanized for livestock depredation (e.g., AM13, Figure 5).

During 2011–2014, prey remains were removed from cougar caches (*n* = 12) in undesirable locations and no aversive conditioning was done. In all cases, cougars returned to investigate the area. In 2 cases, the cougar did not leave the area and killed another small prey item (raccoons) in the vicinity of the original kill. In the remaining 10 cases, the cougar left the area and made its next kill away from human‐developed areas (in open space) but did continue to use developed areas.

## 5 DISCUSSION

We presented data from a long‐term study of cougars utilizing the urban Front Range of Colorado describing how cougars use urban areas, the timing of use, predation on domestic animals, and the potential for aversive conditioning to change cougar behaviors. Although reports of cougars using urban areas are becoming more common, few studies have examined these dynamics. Our results are species‐specific, but are likely broadly applicable to large obligate carnivore species that utilize the wildland**–**urban interface and are bound to interact with humans. Little is known about cougar conflict and depredation on domestic prey because of the difficulty in collecting data on repeat depredation. Conflict and depredation historically have resulted in lethal removal of the offending individual, so the data on such individuals are limited to age and sex. This study is unique in that it was designed to examine human–cougar interaction, so cougars were not lethally removed following conflict, and instead allowed cougars to be monitored for behavioral patterns.

Our findings on cougar space use within the wildland**–**urban interface are similar to those of Kertson et al. (2013), Maletzke et al. (2017), Stoner (2011), and Wang et al. (2017), which are the only other studies that examined cougar populations that interacted within urban areas but also had access to large wildland habitats. Common assumptions are that increasing cougar complaints are associated with increasing subadult and younger age classes (Lambert et al., 2006; Robinson, Wielgus, Cooley, & Cooley, 2008) and that use of exurban habitats is limited to subadults and transients. The results presented here refute these assumptions, demonstrating that all age classes, especially females, utilize these areas. Although all age classes are using these human‐dominated areas, it is females and younger age classes that are more likely to be involved with domestic animal predation conflicts. Kertson et al. (2013) showed a similar pattern of use among all demographic classes utilizing residential areas. Stoner (2011) suggested that it was the maternal females and inefficient hunters (i.e., very young dispersing animals or senescent females) that exploited these riskier urban–wildland habitats to capitalize on food resources.

Areas with high densities of humans are often thought to be low quality habitat for cougars because of increased cougar mortality due to roadkill, lethal removal following depredation involving pets or livestock, and policies favoring cougar removal to maintain human safety (CMGWG, 2005). While these sources of mortality occurred during our study, our data suggest that cougar removals related to depredation and human safety were uncommon relative to the amount of time cougars used these areas and the number of pets taken by cougars, primarily because cougars went undetected. Cougar population density in this area was estimated at 4.1 independent cougars per 100 km^2^, one of the highest reported cougar densities in the literature (Alldredge, Blecha, & Lewis, 2019), indicating that cougars are likely doing very well in these urban habitats. Similar patterns of high carnivore density and human conflict in urban areas have been documented for leopard (*Panthera pardus*) and striped hyena (*Hyaena hayena*) (Athreya, Odden, Linnell, Krishnaswamy, & Karanth, 2013), suggesting the adaptability of carnivores may generally allow them to exploit urban environments despite human conflict and increased risk of mortality.

Our data suggest that the majority (79%) of cougars within the wildland**–**urban interface are avoiding areas with higher housing density. Of the cougars that use higher housing density areas, they generally use these areas at night and leave before morning. Based on this, it would appear that cougars view these areas as risky environments and are avoiding them during periods of higher human activity. Blecha et al. (2018) found that cougars using urban areas were doing so for food acquisition. This suggests that the potential food resource within higher housing density areas is worth the risk, which may be especially true for females with kittens as their energetic demands increase. Females with kittens near urban areas tended to den kittens outside of these areas and make nightly forays into neighborhoods in search of prey. Cougars in urban Western Washington showed similar landscape use patterns of exploiting potential prey resources within urban settings while minimizing the potential for interactions with people (Kertson, Spencer & Grue, 2011; Kertson, Spencer, Marzluff, et al., 2011). Shifting activity patterns to nocturnal periods in exurban areas have also been reported for other large carnivores, such as black bears exploiting urban food resources (Lyons, 2005).

Cougar resource selection showed consistent patterns of habitat use, with the exception of avoidance of the lowest density housing (wildland/rural) relative to exurban habitat. Because wildland and rural habitat are combined, it may be that these areas provide fewer prey resources, especially during the winter, compared with exurban habitat. It is possible that this may be an artifact of lower sampling effort in the western portion of the study area as capture efforts focused more on the exurban eastern edge, but a concurrent study estimating cougar density in this study area suggested a similar distribution of cougars (Alldredge et al., 2019). Regardless, the avoidance of housing was still predictable showing cougar avoidance of humans when in more rural and open areas, and avoidance of suburban and urban areas. Space use patterns of cougars in this landscape demonstrate the highly adaptable behaviors found in many large carnivores as habitat generalists, including black bears (Baruch‐Mordo et al., 2014; Lewis et al., 2015), coyotes (Gehrt et al., 2009; Poessel et al., 2016), and other felids (Burdett et al., 2010; Donovan et al., 2011).

The effect of covariates on the relative risk of cougar incidents was often the opposite of the effect of the covariate on cougar selection. These results demonstrate that if we condition on cougar selection (i.e., if we compare incident locations to where cougars are), the factors driving these occurrences are different from areas that cougars are selecting for. Cougar kills of domestic animals show a similar pattern, especially for domestic cats (Figure 2), which are generally closely associated with housing. Some livestock and large dog conflicts occurred in areas away from housing that are more typically selected by cougars, likely because of overlap with preferred habitats between cougars and free‐ranging livestock. However, this does suggest that animal husbandry practices, especially for dogs, could help reduce conflict (such as not letting dogs roam freely, especially when cougars are active, and covering the top of kennels).

Given the amount of private property that occurred within our study area, cougar harvest was limited. Examining harvest locations (Figures 2 and 3) shows that most harvest occurred in areas where sightings and conflicts were less common. This may indicate that cougar hunting as currently practiced might have limited success as a management strategy to reduce human–cougar interactions, likely due to large amounts of private property limiting common methods of harvest. However, cougars have large home ranges so harvest that occurs outside the wildland**–**urban interface may include animals that also utilize urban areas. Robinson et al. (2008) point out that cougar harvest to reduce population size within small areas is generally ineffective because of high immigration rates as there was a temporal difference between cougar use and human activity.

Being in an undesirable location was the primary type of conflict for female cougars, especially older individuals. All but 2 conflicts associated with undesirable location were reported from a cougar kill of a naturally occurring prey item, demonstrating that the majority of these conflicts involved cougars using higher housing density areas to acquire prey. Moss et al. (2016) in our study area as well as Kertson, Spencer, and Grue (2011) and Robins, Kertson, Faulkner, and Wirsing (2019) all documented a significant use of alternative small‐bodied prey in urban areas compared with studies in wildland areas that documented ungulates as the primary prey for cougars. Undesirable location conflicts occurred across all age classes, but may appear slightly higher for younger cougars (Figure 4), likely representing transient individuals. The potential undesirable location conflict was probably higher for older age females because they regularly used higher housing density areas (based on GPS locations), but older females were rarely seen or reported by the public.

Conflict associated with livestock depredation was more common for male cougars and appeared to be opportunistic, although, on some occasions, an individual would kill multiple livestock over a short time period. Some cougars were euthanized following livestock depredation, so it is difficult to fully assess whether cougars were habituated to preying on livestock. Situations where collared cougars could not be recaptured following livestock depredation were our only opportunities to gather information on the repeat behavior or frequency of livestock kills. Information from cougars that killed livestock that could not be recaptured revealed an opportunistic use of livestock based on long time intervals between these events and did not support habituation to preying on livestock. Cougars likely encountered livestock frequently but infrequently preyed on livestock suggesting that cougars are selecting against livestock, which can be seen with older age classes that infrequently killed livestock. Torres et al. (1996) also found male cougars preyed on livestock significantly more than females in California.

Assessing depredation of pets (cats and dogs) from conflict reports would suggest that these events were rare. Data from California from 1972 to 1995 also suggested pet depredation was relatively uncommon compared with predation on livestock based on permits issued in response to a complaint (Torres et al., 1996). However, pet depredation by cougars based on kill site investigations in our study suggests that this is more common than indicated from conflict reports. This is likely because the small body mass of pets allow prey to be easily cached or moved from the property by cougars and thus rarely discovered by pet owners. For the Front Range of Colorado, on average, we estimated that 4% of a cougar's annual prey (individual kills) was domestic species, primarily small pets. In general, pet depredation appeared to be opportunistic, but two cougars regularly killed pets. We documented, from sampled GPS feeding sites, a 4‐year‐old male that killed 7 dogs over a 14‐month interval and a 2‐year‐old female that killed 8 cats and 2 dogs over a 14‐month interval. These two instances represent minimums because these numbers only represent kill sites that were sampled.

Housing density was the best predictor variable associated with cougars feeding on domestic animals given a sampled feeding event which was expected because of the strong association between domestic animals and houses. There was some evidence that calendar day was a factor influencing cougar use of domestic animals, as the proportion of domestic prey found in feeding sites increased slightly during May (Table 3). Cougars during this study also increased use of higher housing density areas during May, coinciding with an increased use of smaller nonungulate prey (Blecha et al., 2018). Housing density is also a good proxy for the spatial availability of small domestic prey, especially outdoor and feral cats (Blecha, 2015), the primary domestic prey item found in this study. Domestic prey items were more likely to be present at the feeding sites of female cougars, with lower use of domestic pets as females age. Overall, to reduce conflicts associated with cougars killing pets, we recommend managing for an older age class cougar population. Other studies only provided cougar demographic relationships based on conflicts reported to agencies (Aune, 1991; Tiechman et al., 2013; Torres et al., 1996) or the conflict‐related mortality events of collared cougars (Stoner, 2011; Thompson et al., 2014). Future studies should investigate real versus perceived domestic predation frequency using GPS collar sampling methods of feeding sites.

Aversive conditioning within this study was generally ineffective for altering cougar use of urban areas or other undesirable locations, which is likely a result of how treatments were applied because of logistical constraints and cougar behavior. In general, aversive treatments on cougars in undesirable locations were done on cougars returning to their kill of a naturally occurring prey item they made the previous night in a populated area. Similarly, aversive conditioning of cougars following depredation of domestic animals was conducted as the individual returned to their cache, generally in high‐quality cougar habitat. In these situations, the offending cougar had already received a reward (food) for the behavior that we were attempting to condition against. Other data presented here would also suggest that cougars likely utilize these undesirable locations regularly for acquiring resources or kill domestic pets and go undetected in both situations. In ideal circumstances, aversive conditioning would occur at the point in time the undesired behavior is initiated (immediately as they enter the poor location or right as the attack on livestock is initiated). With current GPS technology and real‐time data, it is conceivable that aversive conditioning could be applied as cougars enter these areas or approach livestock, but this would require a huge effort and provides no reasonable long‐term management applications as all cougars would need to have GPS collars.

Although not conclusive, our data suggest that cougars were not habituated to these undesired behaviors that result in conflict events, but were using available resources opportunistically or for foraging opportunities as others were limited. Certainly some cougars were removed after repeated livestock depredations, suggesting habituation. However, cougars that could not be removed after repeated livestock depredation showed a tendency to go back to naturally occurring prey and utilized livestock only opportunistically. Similarly, some cougars used higher housing density areas with some regularity but this may have been driven by food resources. In this area, cougars have been shown to have higher avoidance of human populated areas following feeding events and decreased avoidance as time increases since their last feeding event (Blecha et al., 2018). Most of the higher housing density areas within our study (within cities) had stable and consistent naturally occurring prey including deer, raccoons, and rabbits (Blecha, 2015). These factors only increase the difficulty of applying aversive conditioning techniques within the wildland**–**urban interface.

Removal of cached kills from undesirable locations proved somewhat effective as a means of getting a cougar to leave an urban area, especially when more prey was not immediately available in the area. Cougars tended to leave the area and hunt elsewhere except when raccoons were immediately available. It is possible that cache removal could have long‐term effects on cougars, as these areas would be inefficient for cougars to acquire needed resources, although it is doubtful that managers could find and remove enough carcasses (i.e., those cached by unmarked cougars) to have the desired effect. Removal of livestock kills could have similar effects and cause cougars to leave the area and search for prey elsewhere but only if remaining livestock are unavailable.

Although aversive conditioning appears to be relatively ineffective, there may be hidden benefits that make the effort worthwhile. In all of our efforts to aversively condition cougars, it is likely that we reinforced the idea to cougars that humans present a risk and should be avoided. In many exurban areas of the Front Range, it is becoming more and more common for people to report cougars laying near roads or houses in broad daylight, seemingly unconcerned about humans passing by. In some cases, people can even stop to take pictures of such cougars. This is in contrast to remote areas of Colorado where people rarely see cougars. This apparent bold behavior or habituation to human activity may not be desirable and could lead to increased conflicts. Similar phenomena of increased boldness have been documented for other carnivore species in urban settings, such as brown bears (*Ursus arctos*; Fernandez‐Gil, 2014) and coyotes (Baker & Timm, 1998; Timm, Baker, Bennett, & Coolahan, 2004), including coyotes in Colorado (Breck et al., 2019). It has been postulated that historically intense human persecution of some species selected against bolder individuals and that there has been a recent release from this selective pressure in human‐dominated landscapes that reduce hunting opportunities, thus allowing bolder, more aggressive individuals to thrive in these riskier urban environments (Martinez‐Abrain, Jimenez, & Oro, 2018). There is some question whether such differences in behavioral traits are a result of phenotypic plasticity or of intrinsic differences (Miranda, Schielzeth, Sonntag, & Partecke, 2013). Regardless, carnivore species do appear to be bolder and more aggressive in urban settings and these phenomena correlate well with increasing cougar sightings and conflict that has been observed in the Front Range of Colorado and other urban settings in the West. Given this trend observed across carnivore species, it seems that aversive conditioning of cougars in higher housing density areas could provide indirect benefits in continuing to instill the fear of humans in animals occupying such areas. It may even be beneficial to actively haze cougars that are seen in these settings, that appear to be overly comfortable around human activity, or that exhibit little or no fear of humans.

Cougar characteristics and circumstances surrounding conflict situations were highly variable and did not show consistent patterns. In some situations, an individual would repeat conflict behaviors over a short time period but then stop exhibiting these behaviors and utilize the wildland**–**urban interface for long periods of time without conflict. In other situations, individual cougars were removed after repeat conflict behaviors, resolving issues within certain areas. Individual variation in cougar utilization of human‐dominated landscapes and behavior is not unique to this study (Aune, 1991; Kertson et al., 2013; Riley & Aune, 1997; Robins et al., 2019; Sweanor, Logan, Bauer, Millsap, & Boyce, 2008). Given this variation, it would appear that cougar conflict management should be focused on individual cougars for each unique set of circumstances. Our data also support the conclusions of Kertson et al. (2013) suggesting that human–cougar interactions are a function of individual behavior (learned and innate) and circumstances and that managing for and maintaining an older age structure of cougars in the wildland**–**urban setting would be beneficial.

The coincidence of high cougar density, and extensive use of higher housing density areas and rapid human expansion along the Colorado Front Range, has created a situation where the potential for human–cougar interactions and conflicts is high. However, the realized level of this interaction is relatively low compared with the potential, suggesting that cougars can coexist with people reasonably well. Kertson et al. (2013) came to the same conclusion in an urban setting in western Washington. However, there are steps that people can take to help prevent or limit conflict and promote coexistence in the future. First, it is important to educate the public on cougar behavior and habits in these areas so that people understand what cougars are doing and what they can do to reduce future conflict. Maintaining large (>2,000 ha) open space or natural areas with ample prey and cover for cougars within the urban habitat matrix will provide areas for cougars with limited human activity. We also recommend educating the public about living with cougars, including protecting livestock and pets from cougars and manipulating urban habitats to limit attractions for prey species and limiting hiding cover. People are emotionally attached to hobby livestock and pets, and cougar interactions with these animals generally result in a negative outcome for the cougar even though it is engaging in a natural activity and in many situations is in quality cougar habitat. Therefore, better animal husbandry will help limit cougar conflict and negative attitudes toward coexisting with cougars (CMGWG, 2005). As a final step, we would recommend habitat manipulations within higher housing density areas or areas where cougar presence is not desirable, including green belts within these areas. Habitat manipulations should be directed toward limiting hiding cover for cougars and, more importantly, limiting habitats that are directly beneficial for prey species. Our data presented here and from Moss et al. (2016) and Blecha et al. (2018) strongly suggest that cougars are utilizing these areas to take advantage of alternate prey species, and limiting prey in these areas should limit the motivation of cougars to use these risky habitats.

## CONFLICT OF INTEREST

The authors declare that they have no conflict of interest.

## AUTHOR CONTRIBUTIONS

M.W.A. designed the study, collected field data, analyzed data, and wrote the manuscript. F.E.B. analyzed data and wrote the manuscript. K.A.B. collected field data, analyzed data, and wrote the manuscript. All authors gave final approval for publication.

## ETHICAL APPROVAL

This research complies with the laws of the country in which it was performed. Capture and handling of animals was approved by the institutional animal care and use committee (CPW ACUC ACUC 01‐2007 and 16‐2008).

## Data Availability

The raw data supporting this research are openly available from the Dryad data archive https://doi.org/10.5061/dryad.bt3ng2j.

## References

[ece35559-bib-0001] Alldredge, M. W. , Blecha, T. , & Lewis, J. (2019). Less invasive monitoring of cougars in Colorado's Front Range: An evaluation and review. Wildlife Society Bulletin, 43, 223–230.

[ece35559-bib-0002] Anderson, C. R. , & Lindzey, F. G. (2006). Estimating cougar predation rates from GPS location clusters. Journal of Wildlife Management, 67, 307–316.

[ece35559-bib-0003] Anderson, C. R. J. , Lindzey, F. , Knopff, K. H. , Jalkotzy, M. F. , & Boyce, M. S. (2010). Cougar management in North America In Hornocker M., & Negri S. (Eds.), Cougar: Ecology and Conservation (pp. 41–54). Chicago, IL: University of Chicago Press.

[ece35559-bib-0004] Apker, J. A. , Updike, D. , & Holdermann, D. (2011). Strategies to manage cougar-human interactions In Jenks J. A. (Ed.), Managing cougars in North America (pp. 145–164). Utah State University, Logan, Utah, USA: Jack H. Berryman Institute.

[ece35559-bib-0005] Athreya, V. , Odden, M. , Linnell, J. D. C. , Krishnaswamy, J. , & Karanth, U. (2013). Big cats in our backyards: Persistence of large carnivores in a human dominated landscape in India. PLoS ONE, 8, e57872 10.1371/journal.pone.0057872 23483933 PMC3590292

[ece35559-bib-0006] Aune, K. E. (1991). Increasing mountain lion populations and human‐mountain lion interactions in Montana In Braun C. S. (Ed.), Proceedings of the Mountain Lion‐Human Interaction Symposium and Workshop (pp. 86–94). Denver, CO: Colorado Division of Wildlife.

[ece35559-bib-0007] Avgar, T. , Lele, S. R. , Keim, J. L. , & Boyce, M. S. (2017). Relative selection strength: Quantifying effect size in habitat-and step-selection inference. Ecology and evolution, 7(14), 5322–5330. 28770070 10.1002/ece3.3122 PMC5528224

[ece35559-bib-0008] Baker, R. O. , & Timm, R. M. (1998). Management of conflicts between urban coyotes and humans in southern California In Proceedings of the Vertebrate Pest Conference (vol. 18, pp. 299–312).

[ece35559-bib-0009] Baruch‐Mordo, S. , Wilson, K. R. , Lewis, D. L. , Broderick, J. , Mao, J. S. , & Breck, S. W. (2014). Stochasticity in natural forage production affects use of urban areas by black bears: Implications to management of human‐bear conflicts. PLoS ONE, 9, e85112. 24416350 10.1371/journal.pone.0085122 PMC3885671

[ece35559-bib-0010] Bateman, P. W. , & Fleming, P. A. (2012). Big city life: Carnivores in urban environments. Journal of Zoology, 287, 1–23.

[ece35559-bib-0011] Beckman, J. P. , Lackey, C. W. , & Berger, J. (2004). Evaluation of deterrent techniques and dogs to alter behavior of “nuisance” black bears. Wildlife Society Bulletin, 32, 1141–1146.

[ece35559-bib-0012] Beier, P. (1991). Cougar attacks on humans in the United States and Canada. Wildlife Society Bulletin, 19, 403–412.

[ece35559-bib-0013] Beier, P. , & Barrett, R. H. (1993). The cougar in the Santa Ana Mountain Range, California. Final Report, Orange County Cooperative Mountain Lion Study, University of California Berkley, 104 p.

[ece35559-bib-0014] Beier, P. , Riley, S. P. D. , & Sauvajot, R. M. (2010). Mountain lions (*Puma concolor*) In Gehrt S. D., Riley S. P. D., & Cypher B. (Eds.), Urban carnivores: Ecology, conflict, and conservation (pp. 177–189). Baltimore, MD: John Hopkins University Press.

[ece35559-bib-0015] Blecha, K. A. (2015). Risk‐reward tradeoffs in the foraging strategy of cougar (*Puma concolor*): Prey distribution, anthropogenic development, and patch selection. Thesis, Colorado State University, Fort Collins, CO.

[ece35559-bib-0016] Blecha, K. A. , & Alldredge, M. W. (2015). Improvements on GPS location cluster analysis for the prediction of large carnivore feeding activities: Ground‐truth detection probability and inclusion of activity sensor measures. PLoS ONE, 10, e0138915 10.1371/journal.pone.0138915 26398546 PMC4580633

[ece35559-bib-0017] Blecha, K. A. , Boone, R. B. , & Alldredge, M. W. (2018). Hunger mediates apex predator's risk avoidance response in wildland–urban interface. Journal of Animal Ecology, 87, 609–622. 10.1111/1365-2656.12801 29380374

[ece35559-bib-0018] Breck, S. W. , Poessel, S. A. , Mahoney, P. , & Young, J. K. (2019). The intrepid urban coyote: A comparison of bold and exploratory behavior in coyotes from urban and rural environments.. Scientific Reports: https://www.nature.com/articles/s41598-019-38543-5 10.1038/s41598-019-38543-5 PMC6376053 30765777

[ece35559-bib-0019] Buderman, F. E. , Hooten, M. B. , Alldredge, M. W. , Hanks, E. M. , & Ivan, J. S. (2018). Time‐varying predatory behavior is primary predictor of fine‐scale movement of wildland‐urban cougars. Movement Ecology, 6, 22. 30410764 10.1186/s40462-018-0140-6 PMC6214169

[ece35559-bib-0020] Burdett, C. L. , Crooks, K. R. , Theobald, D. M. , Wilson, K. R. , Boydston, E. E. , Lyren, L. A. , … Boyce, W. M. (2010). Interfacing models of wildlife habitat and human development to predict the future distribution of puma habitat. Ecosphere, 1, 1–21.

[ece35559-bib-0021] C.R.S. § 33‐3‐104 (Lexis Advance through all Laws passed during the 2018 Legislative Session). Colorado Revised Statutes: Parks and Wildlife, Wildlife, Damage by Wildlife, General Provisions, State shall be liable‐when.

[ece35559-bib-0022] Cougar Management Guidelines Working Group (2005). Cougar management guidelines, 1st ed. Bainbridge Island, WA: Wild Futures.

[ece35559-bib-0023] Dickson, B. G. , & Beier, P. (2002). Home range and habitat selection by adult cougars in southern California. Journal of Wildlife Management, 66, 1235–1245. 10.2307/3802956

[ece35559-bib-0024] Donovan, T. M. , Freeman, M. , Abouelezz, H. , Royar, K. , Howard, A. , & Mickey, R. (2011). Quantifying home range requirements for bobcats (*Lynx rufus*) in Vermont. Biological Conservation, 144, 2799–2809.

[ece35559-bib-0025] Ellis, E. C. K. , Goldewijk, K. , Siebert, S. , Lightman, D. , & Ramankutty, N. (2010). Anthropogenic transformation of the biomes, 1700 to 2000. Global Ecology and Biogeography, 19, 589–606. 10.1111/j.1466-8238.2010.00540.x

[ece35559-bib-0026] Erickson, M. E. , Found‐Jackson, C. , & Boyce, M. S. (2014). Using latent selection difference to model persistence in a declining population. PLoS ONE, 9, e98126. 24866172 10.1371/journal.pone.0098126 PMC4035306

[ece35559-bib-0027] Fernandez‐Gil, A. (2014). Osos y lobos: Comportamiento y conservacion de los grandes carnivoros en la Cordillera Cantobrica. Oviedo, Spain: Calecha Ediciones S.L.

[ece35559-bib-0028] Fithzugh, E. L. , Kenyon, M. W. , & Etling, K. (2003). Lessening the impact of a cougar attack on a human In Proceedings of the Seventh Cougar Workshop, Jackson, Wyoming, USA.

[ece35559-bib-0029] Gehrt, S. D. , Anchor, C. , & White, L. A. (2009). Home range and landscape use of coyotes in a metropolitan landscape: Conflict of coexistence. Journal of Mammalogy, 90, 1045–1057.

[ece35559-bib-0030] Gehrt, S. D. , Riley, S. P. D. , & Cypher, B. L. (2010). Urban carnivores: Ecology, conflict, and conservation. Baltimore, MD: Johns Hopkins University Press.

[ece35559-bib-0031] Halfpenny, J. C. , Sanders, M. R. , & McGrath, K. A. (1991). Human‐lion interactions in Boulder County, Colorado: past, present, and future In Braun C. S. (Ed.), Proceedings of the Mountain Lion‐Human Interaction Symposium and Workshop (pp. 10–16). Denver, CO: Colorado Division of Wildlife.

[ece35559-bib-0032] Hansen, A. J. , Knight, R. L. , Marzluff, J. M. , Powell, S. , Brown, K. , Gude, P. H. , & Jones, K. (2005). Effects of exurban development on biodiversity: Patterns, mechanisms, and research needs. Ecological Applications, 15, 1893–1905.

[ece35559-bib-0033] Homstol, L. (2011). Applications of learning theory to human‐bear conflict: The efficacy of aversive conditioning and conditioned taste aversion. Thesis, Department of biological sciences, Edmonton, Alberta.

[ece35559-bib-0034] Iriarte, J. A. , Franklin, W. L. , Johnson, W. E. , & Redford, K. H. (1990). Biogeographic variation of food habits and body size of the America Puma. Oecologia, 85, 185–190. 28312554 10.1007/BF00319400

[ece35559-bib-0035] Johnson, C. J. , Nielsen, S. E. , Merrill, E. H. , McDonald, T. L. , & Boyce, M. S. (2006). Resource selection functions based on use‐availability data: Theoretical motivation and evaluation methods. The Journal of Wildlife Management, 70, 347–357. 10.2193/0022-541X(2006)70[347:RSFBOU]2.0.CO;2

[ece35559-bib-0036] Kertson, B. N. , Spencer, R. D. , & Grue, C. E. (2011). Cougar prey use in a wildland‐urban environment in western Washington. Northwestern Naturalist, 92, 175–185.

[ece35559-bib-0037] Kertson, B. N. , Spencer, R. D. , & Grue, C. E. (2013). Demographic influences on cougar residential use and interactions with people in western Washington. Journal of Mammalogy, 94, 269–281.

[ece35559-bib-0038] Kertson, B. N. , Spencer, R. D. , Marzluff, J. M. , Hepinstall‐Cymerman, J. , & Grue, C. E. (2011). Cougar space use and movements in the wildland‐urban landscape of western Washington. Ecological Applications, 21, 2866–2881.

[ece35559-bib-0039] Knopff, K. H. , Knopff, A. A. , & Boyce, M. S. (2010). Scavenging makes cougars succesptible to snaring at wolf bait-stations. Journal of Wildlife Management, 74, 644–653.

[ece35559-bib-0040] Korn, E. L. , & Graubard, B. I. (1998). Confidence intervals for proportions with small expected number of positive counts estimated from survey data. Survey Methodology, 24, 193–201.

[ece35559-bib-0041] Lambert, C. M. S. , Wielgus, R. B. , Robinson, H. S. , Katnik, D. D. , Cruickshank, H. S. , Clarke, R. , & Almack, J. (2006). Cougar population dynamics and viability in the Pacific Northwest. Journal of Wildlife Management, 70, 246–254.

[ece35559-bib-0042] Latham, A. D. M. , Latham, M. C. , & Boyce, M. S. (2011). Habitat selection and spatial relationships of black bears (*Ursus americanus*) with woodland caribou (*Rangifer tarandus caribou*) in northeastern Alberta. Canadian Journal of Zoology, 89, 267–277.

[ece35559-bib-0043] Leigh, J. (2007). Effects of aversive conditioning on behavior of nuisance Louisiana black bears. Louisiana State University Master's thesis.

[ece35559-bib-0044] Lele, S. R. , Merrill, E. H. , Keim, J. , & Boyce, M. S. (2013). Selection, use, choice and occupancy: Clarifying concepts in resource selection studies. Journal of Animal Ecology, 82, 1183–1191. 24499379 10.1111/1365-2656.12141

[ece35559-bib-0045] Lendrum, P. E. , Northrup, J. M. , Anderson, C. R. , Liston, G. E. , Aldridge, C. L. , Crooks, K. R. , & Wittemyer, G. (2018). Predation risk across a dynamic landscape: Effects of anthropogenic land use, natural landscape features, and prey distribution. Landscape Ecology, 33, 157–170.

[ece35559-bib-0046] Lewis, D. L. , Baruch‐Mordo, S. , Wilson, K. R. , Breck, S. W. , Mao, J. S. , & Broderick, J. (2015). Foraging ecology of black bears in urban environments: Guidance for human‐bear conflict mitigation. Ecosphere, 6, 141 10.1890/ES15-00137.1

[ece35559-bib-0047] Lyons, A. J. (2005). Activity patterns of urban American black bears in the San Gabriel Mountains of southern California. Ursus, 16, 255–262.

[ece35559-bib-0048] Maletzke, B. , Kertson, B. , Swanson, M. , Koehler, G. , Beausoleil, R. , Wielgus, R. , & Cooley, H. (2017). Cougar response to a gradient of human development. Ecosphere, 8(7), e01828 10.1002/ecs2.1828

[ece35559-bib-0049] Manly, B. F. L. , McDonald, L. , Thomas, D. L. , McDonald, T. L. , & Erickson, W. P. (2007). Resource selection by animals: Statistical design and analysis for field studies. Berlin, Germany: Springer Science & Business Media.

[ece35559-bib-0050] Martinez‐Abrain, A. , Jimenez, J. , & Oro, D. (2018). Pax Romana: ‘refuge abandonment’ and spread of fearless behavior in a reconciling world. Animal Conservation, 22, 3–13.

[ece35559-bib-0051] Mattson, D. J. (2007). Mountain lions of the Flagstaff Uplands. 2003–2006 Progress report. USGS Open File Report 2007–1062.

[ece35559-bib-0052] Mazur, R. L. (2010). Does aversive conditioning reduce human‐black bear conflict? Journal of Wildlife Management, 74, 48–54.

[ece35559-bib-0053] McBride, R. , Jansen, D. K. , McBride, R. , & Schulze, S. R. (2005). Aversive conditioning of Florida panthers by combining painful experiences with instinctively threatening sounds In Proceedings of the 8th Mountain Lion Workshop (p. 136).

[ece35559-bib-0054] McCarthy, T. M. , & Seavoy, R. J. (1994). Reducing nonsport losses attributable to food conditioning: Human and bear behavior modification in an urban environment. International Conference on Bear Research and Management, 9, 75–84.

[ece35559-bib-0055] Miranda, A. C. , Schielzeth, H. , Sonntag, T. , & Partecke, J. (2013). Urbanization and its effects on personality traits: A result of microevolution or phenotypic plasticity? Global Change Biology, 19, 2634–2644. 23681984 10.1111/gcb.12258

[ece35559-bib-0056] Moss, W. E. , Alldredge, M. W. , & Pauli, J. N. (2016). Quantifying risk and resource use for a large carnivore in an expanding urban‐wildland interface. Journal of Applied Ecology, 53, 371–378.

[ece35559-bib-0057] Murphy, K. , & Ruth, T. K. (2010). Diet and prey selection of a perfect predator In Hornocker M., & Negri S. (Eds.), Cougar: Ecology and conservation (pp. 118–137). Chicago, IL: University of Chicago Press.

[ece35559-bib-0058] Pickett, S. T. A. , Cadenasso, M. L. , Grove, J. M. , Nilon, C. H. , Pouyat, R. V. , Zipperer, W. C. , & Costanza, R. (2001). Urban ecological systems: Linking terrestrial ecological, physical, and socioeconomic components of metropolitan areas. Annual Review of Ecology and Systematics, 32, 127–157.

[ece35559-bib-0059] Poessel, S. A. , Breck, S. W. , & Gese, E. M. (2016). Spatial ecology of coyotes in the Denver metropolitan area: Influence of the urban matrix. Journal of Mammalogy, 97, 1414–1427.

[ece35559-bib-0060] R Core Team (2017). R: A language and environment for statistical computing. Vienna, Austria: R Foundation for Statistical Computing Retrieved from https://www.R-project.org/

[ece35559-bib-0061] Rabinowitz, A. (2010). Forward In Hornocker M., & Negri S. (Eds.), Cougar: Ecology and conservation (pp. viii–x). Chicago, IL: University of Chicago Press.

[ece35559-bib-0062] Rauer, G. , Kaczensky, P. , & Knauer, F. (2003). Experiences with aversive conditioning of habituated brown bears in Austria and other European countries. Ursus, 14, 215–224.

[ece35559-bib-0063] Reiter, D. K. , Brunson, M. W. , & Schmidt, R. H. (1999). Public attitudes toward wildlife damage management and policy. Wildlife Society Bulletin, 27, 746–758.

[ece35559-bib-0064] Riley, S. J. , & Aune, K. E. (1997). Mountain lion‐human and mountain lion‐livestock incidents in Montana In Padley W. D. (Ed.) Proceedings of the Fifth Mountain Lion Workshop (pp. 91). San Diego, CA: California Department of Fish and Game.

[ece35559-bib-0065] Robins, C. W. , Kertson, B. N. , Faulkner, J. R. , & Wirsing, A. J. (2019). Effects of urbanization on cougar foraging ecology along the wildland‐urban gradient of western Washington. Ecosphere, 10(3), e02605 10.1002/ecs2.2605

[ece35559-bib-0066] Robinson, H. S. , Wielgus, R. B. , Cooley, H. S. , & Cooley, S. W. (2008). Sink populations in carnivore management: Cougar demography and immigration in a hunted population. Ecological Applications, 18, 1028–1037. 18536260 10.1890/07-0352.1

[ece35559-bib-0067] Roever, C. L. , Beyer, H. L. , Chase, M. J. , & Aarde, R. J. (2014). The pitfalls of ignoring behaviour when quantifying habitat selection. Diversity and Distributions, 20, 322–333.

[ece35559-bib-0068] Ruth, T. K. (1991). Cougar use in an area of high recreational development in Big Bend National Park, Texas. Thesis, Texas A&M University, College Station, Texas, USA.

[ece35559-bib-0069] Shaw, H. G. (1977). Impacts of mountain lion on mule deer and cattle in northwestern Arizona In Phillips R. L., & Jonkel C. J. (Eds.), Proceedings of the 1975 Predator Symposium (pp. 17–32). Missoula, MT: Montana Forest and Conservation Experiment Station, University of Montana.

[ece35559-bib-0070] Shivik, J. A. , & Martin, D. J. (2000). Aversive and disruptive stimulus applications for managing predation In The 9th Wildlife Damage Management Conference Proceedings (vol. 9, pp. 111–119).

[ece35559-bib-0071] Smith, J. A. , Wang, Y. , & Wilmers, C. C. (2016). Spatial characteristics of residential development shift large carnivore prey composition. Journal of Wildlife Management, 80, 1040–1048.

[ece35559-bib-0072] Stoner, D. C. (2011). Ecology and conservation of cougars in the eastern Great Basin: Effects of urbanization, habitat fragmentation, and exploitation. Dissertation, Utah State University, Logan, UT.

[ece35559-bib-0073] Suminski, H. R. (1982). Mountain lion predation on domestic livestock in Nevada In March R. E. (Ed.), Proceedings of the tenth vertebrate pest conference (pp. 62–66). Davis, CA: University of California.

[ece35559-bib-0074] Sweanor, L. L. , Logan, K. A. , Bauer, J. W. , Millsap, B. , & Boyce, W. M. (2008). Puma‐human relationships in Cuyamaca Rancho State Park California. Journal of Wildlife Management, 72, 1076–1084.

[ece35559-bib-0075] Theobald, D. M. (2005). Landscape patterns of exurban growth in the USA from 1980 to 2020. Ecology and Society, 10, 32.

[ece35559-bib-0076] Theobald, D. (2007). LCaP v1. 0: Landscape connectivity and pattern tools for ArcGIS. Fort Collins, CO: Colorado State University.

[ece35559-bib-0077] Theobald, D. M. , Harrison‐Atlas, D. , Monahan, W. B. , & Albano, C. M. (2015). Ecologically‐relevant maps of landforms and physiographic diversity for climate adaptation planning. PLoS ONE, 10, e0143619 10.1371/journal.pone.0143619 26641818 PMC4671541

[ece35559-bib-0078] Thompson, D. J. , Jenks, J. A. , & Fecske, D. M. (2014). Prevalence of human‐caused mortality in unhunted cougar population and potential impacts to management. Wildlife Society Bulletin, 38, 341–347.

[ece35559-bib-0079] Tiechman, K. J. , Cristescu, B. , & Nielsen, S. (2013). Does sex matter? Temporal and spatial patterns of cougar‐human conflict in British Columbia. PLoS ONE, 8, e74663 10.1371/journal.pone.0074663 24040312 PMC3770613

[ece35559-bib-0080] Timm, R. M. , Baker, R. O. , Bennett, J. R. , & Coolahan, C. C. (2004). Coyote attacks: An increasing suburban problem In Transactions North American Wildlife Natural Resource Conference (vol. 69, pp. 67–88).

[ece35559-bib-0081] Torres, S. G. , Mansfield, T. M. , Foley, J. E. , Lupo, T. , & Brinkhaus, A. (1996). Mountain lion and human activity in California: Testing speculations. Wildlife Society Bulletin, 24, 451–460.

[ece35559-bib-0082] van Eeden, L. M. , Eklund, A. , Miller, J. R. B. , Lopez‐Bao, J. V. , Chapron, B. , Cejtin, M. R. , … Treves, A. (2018). Carnivore conservation needs evidence‐based livestock protection. PLoS Biology, 16(9), e2005577 10.1317/journal.pgio.2005577 30226872 PMC6143182

[ece35559-bib-0083] Vitousek, P. M. , Mooney, H. A. , Lubchenco, J. , & Melillo, J. M. (1997). Human domination of Earth's ecosystems. Science, 277, 494–499. 10.1126/science.277.5325.494

[ece35559-bib-0084] Wang, Y. , Smith, J. S. , & Wilmers, C. C. (2017). Residential development alters behavior, movement, and energetics in an apex predator, the puma. PLoS ONE, 12, e0184687 10.1371/journal.pone.0184687 29020087 PMC5636101

[ece35559-bib-0085] Wilmers, C. C. , Wang, Y. , Nickel, B. , Houghtaling, P. , Shakeri, Y. , Allen, M. L. , … Williams, T. (2013). Scale dependent behavioral responses to human development by a large predator, the puma. PLoS ONE, 8, e60590 10.1371/journal.pone.0060590 23613732 PMC3629074

[ece35559-bib-0086] Woodroffe, R. (2000). Predators and people: Using human density to interpret declines of large carnivores. Animal Conservation, 3, 165–173.

[ece35559-bib-0087] Zarco‐Gonzalez, M. M. , & Monroy‐Vilchis, O. (2014). Effectiveness of low‐cost deterrents in decreasing livestock predation by felids: A case in Central Mexico. Animal Conservation, 17, 371–378. 10.1111/acv.12104

